# Perceptions of the MDT towards the health needs and support for grandparents caring for AIDS orphans

**DOI:** 10.4102/hsag.v29i0.2759

**Published:** 2024-10-24

**Authors:** Furaha Akimanimpaye, Million S. Bimerew, Isaiah D. Owoeye, Deliwe R. Phetlhu

**Affiliations:** 1 School of Nursing, Faculty of Community Health Sciences, University of the Western Cape, Cape Town, South Africa; 2 Department of Nursing Science, Faculty of Health Care Sciences, Sefako Makgatho Health Sciences University, Ga-Rankuwa, Pretoria, South Africa

**Keywords:** AIDS, grandparents, health need, support, orphans

## Abstract

**Background:**

The acquired immunodeficiency syndrome (AIDS) crisis has orphaned many children, shifting care giving duties to grandparents. This has challenged the health of the grandparents, underscoring the need for support in caring for both the grandparents and the children orphaned due to AIDS.

**Aim:**

This study investigated the multidisciplinary team’s (MDT) perceptions of health needs and support for grandparents caring for children orphaned due to AIDS.

**Setting:**

The study took place within Metropolitan area in the Western Cape Province, South Africa.

**Methods:**

A cross-sectional descriptive survey was used to assess health needs and support for grandparents caring for children orphaned due of AIDS. A sample of 65 MDT members comprising professional nurses, psychologists and social workers participated in the study.

**Results:**

The assessment revealed moderate awareness (mean 2.79) among MDT regarding the health needs of grandparents caring for the orphans. Key concerns were a lack of income (mean = 4.9) and no access to health services (mean = 4.8), with low awareness and involvement in organisational support (mean = 1.8).

**Conclusion:**

Despite an awareness of the health needs and support perceptions among grandparents, there remains a significant gap in the availability of support structures to address these needs. The study highlights the critical necessity for developing robust support systems to enhance the well-being of grandparents, especially those caring for AIDS orphans, ensuring they receive the comprehensive care and assistance required.

**Contribution:**

The study’s findings provide key insights for supporting grandparents who are caring for AIDS orphans via a MDT approach.

## Introduction

The global effect of the acquired immunodeficiency syndrome (AIDS) pandemic has created a disruption in the family system all over the world. Globally, about 630 000 AIDS-related deaths occurred in the year 2022 and the nations are advised to brace up their responsibility to safeguard the health of their citizens as the threatening side of AIDS is forecast by the year 2030 (WHO 2024).

The prevalence of the burden of AIDS in the world was 476 cases per 100,000, although the statistics dropped at a point because of antiretroviral use, but increased again in the last decade (Govender et al. 2021). In the world, there are about 15 million orphans who are with their grandparents because of the death of their parents (Nabunya 2023). There is a steady rise in the statistics of orphans every year (Ahmad & Mohamed 2022). This brings about the situation whereby the orphans are left to depend on their aged grandparents who in turn are not strong both physically and psychologically to assume such a task (Osafo et al. 2017).

In Africa, the impact of AIDS on the population has been well-documented (Govender et al. 2021). The impact is reflected in the lives of mothers living with AIDS as the prevalence is more among the female gender (Fauk et al. 2017). The death of parents in Africa often leaves their children in the care of their closest relatives (Likoko et al. 2023). This is not typical of Africa alone as children orphaned because of AIDS are emerging concept with 140 million documented globally and 7.5 million recorded in the United States (Mthembu, Myburgh & Poggenpoel 2020). Furthermore, Africa has documented 15.4 million orphans who have lost their parents to AIDS (Misore 2023). Approximately, 60% of these orphans are taken in by their grandparents following the death of their parents (Mhaka-Mutepfa 2018). This places the responsibility of caring for these orphans on grandparents, who already have their own health needs to attend to, thus increasing the grandparents’ responsibilities. Moreover, these grandparents face numerous challenges, including financial strain, health issues related to ageing, and limited access to resources and social support (Taylor et al. 2017).

The prevalence of AIDS is rising in South Africa significantly affecting family dynamics (Govender et al. 2021). As is the case elsewhere, the loss of parents to this epidemic frequently leads to children becoming orphans, with grandparents assuming the caregiving role (Likoko et al. 2023). Although grandparenting is a contextual issue in South Africa, the trend of grandparents taking over the responsibility as caregivers is evident from the early period when the AIDS impact become pronounced (Ahmad & Mohamed 2022; Likoko et al. 2023; Phetlhu &Watson 2014). In the year 2021, approximately 960 000 orphans in South Africa were attributed to the deaths of parents from HIV and AIDS-related causes (Gutura & Khosa 2023). South Africa leads statistics on most incidences of children who have lost their parents to AIDS, with almost half of the orphans being because of AIDS-associated occurrence (Mtshali 2016).

The burden of caring for vulnerable children orphaned because of AIDS poses significant health risks because of undue stress that could compromise body agility and the human immune system. This responsibility, coupled with the inherent challenges of caregiving, places a considerable strain on their biopsychosocial needs, which include basic needs and health needs (Lazaro, Walker & Robson 2023). Challenges in providing care for themselves and support their grandchildren often revolve around their financial capabilities leading to inability to cope with daily financial demand as a result of financial stress occasioned by additional responsibilities (Jennings, Farrell & Kobayashi 2021).

Assuming dual roles in caring for both themselves and the orphans, grandparents often neglect their own health needs, necessitating a comprehensive examination of their health status and available support systems. Thus, there is a need for a comprehensive support from multidisciplinary team (MDT). A lack of the necessary support towards grandparents in their caring role may lead to physical, emotional and psychological distress, which can result in depression, anxiety, financial and social stress, burnout and a decreased quality of life (Mthembu et al. 2020). Recent studies emphasised the need for comprehensive care approaches that provide them with adequate intervention and support (Nabunya et al. 2023).

Assisting grandparents in caring for children orphaned because of AIDS is crucial for addressing their health needs; however, this is not limited to financial aid but include equitable resource distribution and access to healthcare facilities, among others (Nabunya 2023). Although a study explored the understanding of social support among children orphaned because of AIDS (Ahmad & Mohamed 2022), there exist insufficient data regarding support systems tailored specifically to grandparents entrusted with their care (Lazaro et al. 2023). Given the significance of the health needs and available support for grandparents caring for children orphaned because of AIDS. Likoko et al. (2023) stress the imperative of implementing intervention strategies to tackle the health challenges faced by grandparents, highlighting the necessity for comprehensive support.

In South Africa, professional nurses, psychologists and social workers often operate with varying levels of expertise, resulting in a fragmented delivery of essential resources and support. This disjointed approach contributes to insufficient assistance for grandparents caring for children orphaned because of AIDS, which has enduring detrimental effects on these vulnerable populations. However, the MDTs’ approach, which could be accessed through an enabling policy in health facilities, comprising these health professionals, is preferable as it tackles the challenges of fragmentation in care to the grandparents. To address these challenges, it is imperative to adopt a more organised and integrated approach, where the healthcare professionals are saddled with the role of managing the health needs of the grandparents in a wholistic manner. Such an approach would ensure comprehensive support for children orphaned because of AIDS and their grandparents, safeguarding them from risks such as poverty, social isolation and unmet healthcare needs (Gutura & Khosa 2024; Mtshali 2016). Strengthening coordination and collaboration among MDT members is crucial to enhancing the overall well-being and care outcomes for these families.

Adopting a collaborative approach that engages a MDT is vital. Psychologists can tackle emotional and psychological distress, nurses can attend to physical health requirements, and social workers can offer crucial social support, resources and guidance in navigating the healthcare system. Grandparenting health needs and available support are critical in the care of children orphaned because of AIDS. According to Likoko et al. (2023), prompt attention to interventional strategies to ameliorate the grandparent’s health challenges is needed through adequate support. This combined effort plays a pivotal role in substantially enhancing the overall health and well-being of grandparents who shoulder the responsibility of caring for children orphaned AIDS (Lazaro et al. 2023).

The existing support mechanisms for grandparents, such as pension schemes and welfare grants, are considered insufficient (Jennings et al. 2021). While the literature acknowledges the psychosocial needs of these grandparents (Lazaro et al. 2023), there remains a significant gap in studies specifically addressing their health needs and the support available to them. Notably, the South African government lacks tailored programmes that extend beyond financial assistance to support these grandparents (Soganga & Kang’ethe 2023; Taylor et al. 2017). To address this gap, this study aimed to assess the perceptions of a MDT regarding the health needs and support for grandparents caring for children orphaned because of AIDS in the Western Cape province of South Africa.

### Problem statement

Grandparents caring for children orphaned because of AIDS are faced with a variety of challenges as a result of the caring responsibilities that bring a multitude of challenges, encompassing resource deficits, the strain of caregiving and a lack of adequate support (Kalomo & Liao 2018). However, the literature indicates that grandparents are frequently overlooked and not adequately supported in their caregiving responsibilities, which can have negative impact on their overall health (Fauk et al. 2017). Thus, tackling their health needs necessitates a collaborative approach from MDTs to ensure a comprehensive care plan that addresses their social, medical and psychological requirements (Damian, Mashau &Tugli 2019). In South Africa, despite the good work performed by the MDT in various healthcare settings and communities, there is a lack of evidence on how grandparents caring for children orphaned because of AIDS are broadly supported and the services they provide to them, hence a need to ascertain their health needs. An understanding of the health needs and the support given to grandparents caring for children orphaned because of AIDS might contribute to understanding the strategies required to enhance the support given to them.

### Study purpose

The study aimed to investigate the MDT’s perceptions of health needs and support for grandparents caring for children orphaned because of AIDS in the Western Cape province, South Africa.

## Research methods and design

### Study design

This study utilised a quantitative cross-sectional survey to evaluate the health needs and available support for grandparents caring for children orphaned because of AIDS in the Western Cape province, South Africa. The study is guided by Clark’s (2003) dimensions model of community health nursing, the research aimed to investigate the MDT’s perceptions of these grandparents’ health needs and support systems. This model offers a holistic approach, addressing the physical, social, emotional and healthcare system requirements of grandparents responsible for caring for children orphaned because of AIDS.

### Setting

The research was carried out in the Western Cape province, South Africa. The study focused on two districts: the City Metropolitan Municipality encompassing the urban area and the Municipality, encompassing the rural farming area. The aforementioned study setting was chosen because they have significant levels of HIV infection, along with high rates of poverty, limited education access and elevated crime rates, leading to a substantial number of orphans within their communities (Simbayi et al. 2019).

### Population and sampling

The sample comprised 65 MDT health professionals, comprising professional nurses, psychologists and social workers, all active within the selected study area. The researchers employed a two-stage sampling technique. Initially, a stratified sampling approach was utilised to select 13 community health centres, comprising 5 clinics in Khayelitsha, 5 clinics in Mitchell’s Plain and 3 clinics in Grabow. Following this, within each clinic, the sample size was determined using an all-inclusive sampling method because of the limited size of the population. However, to ensure the validity of the sample personnel who were on leave during the data collection period were excluded. This method was deemed suitable for the study under the presumption that participants possessing pertinent information crucial for the study were scarce, thus excluding any would further diminish the sample pool.

### Data collection

Self-administered questionnaire served as the research instrument in the study. The researcher developed the instrument in English. The researchers formulated the questionnaires by drawing upon evidence from best practise standards in comparison to the prevailing practises in healthcare (Haber 2019). These standards were tailored to address the specific aims, design, and context of this study, particularly focusing on the health needs and provision of support to grandparents caring for children orphaned because of AIDS.

The questionnaire incorporated a combination of both fixed-response and open-ended items, along with skip patterns. While its primary objective was to gather quantifiable data to depict the health needs and support requirements of grandparents caring for children orphaned because of AIDS, it also allowed for the collection of narrative responses. The questionnaire was structured into three sections. Section A focused on the demographic information of the respondents, with assurance of anonymity and confidentiality given by the researchers through the exclusion of respondent’s names. Sections B and C assessed the health needs and the support available to grandparents.

The reliability of the questionnaire was tested before being administered. The questionnaire exhibited a Cronbach coefficient of 0.754, signifying strong internal consistency for the instrument. Before initiating data collection, the researchers conducted a pilot study involving seven participants who exhibited characteristics comparable to those of the study population. After analysing the results of this pilot study, the research team proposed particular modifications to enhance the questionnaire’s structure. These adjustments involved consolidating related themes to improve the overall coherence and logical progression of the surveys. Subsequently, these enhancements were incorporated into the survey instrument.

During the data collection period from June to August 2018, researchers personally distributed questionnaires to participants in sealed envelopes, ensuring confidentiality. Each participant, having agreed to volunteer for the study, received the questionnaire in their respective offices along with a consent form to sign. A specific due date for the return of the questionnaires was provided and adhered to by the participants, ensuring an organised and timely data collection process.

### Data analysis

Data were analysed using SPSS version 29. The responses ‘agree’ and ‘strongly agree’ were combined into a single ‘agree’ category. Four statements from the scale were recoded to reverse their wording: ‘Lack of income for survival can be a contributing factor to health problems among grandparents caring for AIDS orphans’, ‘No access to health services can exacerbate the already compromised health of grandparents caring for AIDS orphans’, ‘Absence of basic needs may cause health problems among grandparents caring for AIDS orphans’, and ‘Lack of social security provision can be the major cause of stress experienced by grandparents caring for AIDS orphans’. Descriptive statistics were used to present the statements on the awareness scale, arranging them from most to least significant.

### Ethical considerations

Approval to conduct this study was duly obtained from the University of the Western Cape Ethics Committee (Reference: HS 16/5/31) and permission was obtained from the various health institutions and social services where the study was conducted. Thereafter, consent for the study was obtained by asking participants to fill and return the consent forms after due explanation of the study. Before participation, respondents were provided with detailed information regarding the study’s nature, objectives, data collection methods and the questionnaire instrument. They were also assured of their voluntary participation and the freedom to withdraw from the study at any point. Furthermore, respondents were guaranteed that all information about them would be treated with confidentiality and would not be utilised for any other purposes without their explicit consent.

## Results

Out of a sample of 65 respondents, 50 completed the questionnaire, resulting in a 77% response rate. The respondents had a mean age of 33.9 years (s.d. = 7.82). The majority of respondents were female (74.0%) and predominantly from urban areas (82.0%). In addition, over half of the respondents (56.0%) (Table 1) held an honours degree.

**TABLE 1:** Sample realisation and demographics.

Items	Responses		Range
	*n*	%	
**Age (mean, s.d.)**	33.9	7.82	23–59
**Gender**			
Female	37	74.0	-
Male	13	26.0	-
**Location**			
Urban	41	82.0	-
Rural	9	18.0	-
**Multidisciplinary team**			
Professional nurse	30	60.0	-
Social worker	13	26.0	-
Psychologist	7	14.0	-
**Educational level**			
Matric	2	4.0	-
Degree	16	32.0	-
Honours	28	56.0	-
Masters	4	8.0	-

s.d., standard deviation.

When assessing the awareness of health needs among MDT regarding the perceived health needs of grandparents, the average mean rating was 2.79. The highest-rated statements were ‘Lack of income for survival can be a contributing factor to health problems among grandparents caring for AIDS orphans’ (mean = 4.9 ± 0.36) and ‘No access to health services can exacerbate the already compromised health of grandparents caring for AIDS orphans’ (mean = 4.8 ± 0.39). Conversely, the lowest-rated statement was ‘I am aware and actively involved in the roles the organisation is playing in provision of support given to grandparents’ (mean = 1.8 ± 0.64). Overall, respondents demonstrated a moderate awareness of the health needs, with a mean score of 2.79 out of 5 (Table 2).

**TABLE 2:** Awareness of health needs.

Statement	Mean (s.d.)		Agreed	
	*n*	%	*n*	%
A lack of income for survival can be a contributing factor to health problem among grandparents caring for AIDS orphans.	4.9	0.36	50	100.0
No access to health services can exacerbate the already compromised health of grandparents caring for AIDS orphans.	4.8	0.39	50	100.0
Absence of basic needs may cause health problems among grandparents caring for AIDS orphans	4.7	0.49	48	96.0
I am aware of the health risk on the grandparents caring for AIDS orphans in general.	4.6	0.71	47	94.0
A lack of social security provision can be the major cause of stress experienced by grandparents caring for AIDS orphans.	4.6	0.57	48	96.0
I can easily identify health problems among grandparent caregivers of AIDS orphans who make use of the clinic where I work.	4.2	1.16	40	80.0
I am aware of urgent needs and problems grandparents caring for AIDS orphans are facing.	4.0	0.93	37	74.0
I already encountered different categories of grandparents caring for AIDS orphans.	4.0	0.93	39	78.0
The health problems of grandparents caring for AIDS orphans are escalating is in our communities.	4.0	0.90	32	64.0
I am aware of the impacts of AIDS on the welfare of grandparents caring for AIDS orphans here in Western province.	3.3	1.10	19	38.0
I am aware of the existing care plan and support services provided by my clinic, to the needy grandparents caring for AIDS orphans.	2.6	1.41	11	22.0
I am aware and actively involved in the roles the organisation is playing in provision of support given to grandparent.	1.8	0.64	0	0.0

Note: Mean 2.79.

AIDS, acquired immunodeficiency syndrome; s.d., standard deviation.

Findings from this study also revealed that MDT healthcare services did not have a professional practitioner dedicated to work and conduct home visits with grandparents caring for children orphaned because of AIDS both in rural and urban communities.

In assessing the available support for grandparents, it was found that more than half of the respondents (59.2%) were unaware of the support contributions, and none of the respondents (0.0%) were actively involved in providing support (Table 3). The qualitative analysis of the statements revealed two key themes: provision of holistic support and activation of referrals. Emotional support and social grants were among the types of assistance mentioned by respondents. In terms of referral activation, some respondents suggested regular clinic visits for grandparents, referrals for adolescents to Counseling Youth and Young People (CYP), and comprehensive medical support through complete screenings.

**TABLE 3:** Perception of available support.

Statement	Frequency	%
**Functions in providing support?**		
Yes	0	0.0
**Evaluation of MDT contribution of support given**		
Don’t know	29	59.2
Not good	18	36.7
Somewhat good	2	4.1
**Support strategies tried**		
No strategies tried	17	34.7
Providing emotional support	16	32.7
Providing referral	15	30.6
Providing social economical support in their residential setting	1	2.0

MDT, multidisciplinary team.

## Discussion of findings

The study found that respondents had an average age of 33.9 years (s.d. = 7.82), with 74.0% being female, which contrasts with Misore’s (2023) findings where 68.5% of health workers were male, and most were aged 40 years and above. These demographic disparities may reflect differences in workforce compositions across regions or health sectors. The higher proportion of younger females in our study suggests a shift towards a more gender-diverse and youthful workforce in the Western Cape province, possibly because of recent recruitment and training efforts. In contrast, Misore’s study revealed an older, predominantly male demographic, indicative of a more traditional workforce composition shaped by historical gender roles and career longevity in certain sectors.

### Awareness of health needs

This study highlights the significant impact of inadequate income on the health of grandparents caring for AIDS orphans. This finding resonates with previous research conducted by Phetlhu and Watson (2014) in the North West province of South Africa, which reported that many grandparents facing the responsibility of caring for children orphaned because of AIDS struggle with unstable income, exacerbating their health challenges. Similarly, Nabunya (2023) conducted a study among adolescents in Uganda, revealing that financial constraints were the primary concern for families headed by grandparents, hindering their ability to meet essential family needs.

Furthermore, this study aligns with findings from Misore (2023) in Kenya, where a significant proportion of caregivers, approximately 58%, were living below the poverty line, making it difficult for them to adequately support their own health needs, let alone those of the orphans in their care. Shaibu (2016) also explored the experiences of Botswana grandparents caring for children orphaned because of AIDS and found that financial limitations were a major barrier to accessing healthcare for the orphans.

Insightfully, a study conducted in South Africa revealed the challenges grandparents face in accessing social grants, with the process often taking up to 12 months to complete. This delay can result in significant financial strain and hardship for grandparents before receiving the much-needed assistance (Gutura & Khosa 2024). Overall, these findings underscore the urgent need for comprehensive support systems and interventions to address the financial challenges faced by grandparents caring for children orphaned because of AIDS, thereby safeguarding both their own health and the well-being of the children in their care.

This study has shed light on the detrimental impact of limited access to healthcare on the already compromised health of grandparents caring for children orphaned because of AIDS. A study conducted in South Africa underscored the urgency of addressing this issue, highlighting that grandparents with poor health were more likely to seek healthcare to address their health concerns. This emphasises the need for comprehensive health programmes aimed at alleviating the health challenges faced by grandparents (Likoko et al. 2023). This finding aligns with research conducted by Taylor et al. (2017) in Australia, where grandparents discussed the significant health implications associated with caring for the orphans left behind by their deceased children. This emphasises the importance of ensuring access to healthcare services for grandparents to support their well-being as they navigate the complexities of caregiving.

Taylor et al. (2017) pointed a need for access to medical intervention programme to addressing health issue among the grandparents. This study is not consistent with a study by Shaibu (2016) among grandmothers caring for children orphaned because of AIDS in Rwanda where impediments to access to health by the grandparents were portrayed to be ignorant on health status and were also forgetful of the appointed date of medical cheque-up. In a study, the grandparents posited that the health issues have deprived them of positive engagement with the care of the orphans. This study is in agreement with another related study by Peterson (2017), which was carried out in the United States among the grandparents, the study revealed that the grandparents had an awareness of the danger that caregiving of the grandchildren had on their health status.

### Support for the grandparents

In this study, more than half of the respondents (29, 59.2%) do not know the contribution of support given to the grandparents and none of the respondents (0, 0.0%) function in providing support for the grandparents. In another study in Kenya, 47% of the health workers do not have the knowledge of existing support to the grandparents AIDS (Misore 2023). This is in agreement with a review which stated that health professionals do not provide support for the overall health and welfare of the grandparents because the emphasis is solely on the orphans (Gutura & Khosa 2024). To corroborate this, another study in South Africa carried out among grandparents revealed there was no structured support mainly for grandparents of children orphaned because of AIDS (Jennings et al. 2021). The study is consistent with a study by Nabunya (2023), conducted among adolescent orphans in Uganda, which documented that the availability of a support network is reported as part of the pivot contributing factors to wellness of the people. The response of the MDT denotes that there may be little or no network of support available for the grandparents caring for the orphans and the health teams are not engaged in provision of service in that regard.

This study revealed a need for provision of holistic support, including financial support, emotional support and social grant for the grandparents. This is in line with a study conducted in South Africa where the grandparents stated that financial issue was the main barrier to caring for the children orphaned because of AIDS and the participants also reported that they did try to seek support but to no avail (Phetlhu & Watson, 2014). This is also in line with a study by Gutura and Khosa (2024) where it was recorded that grandparents caring for orphans of their dead children faced myriads of difficulties among which were financial instability, social exclusion and challenges in sustaining link with the health workers. Physiological and affection issues regarding the care of the orphans could be addressed through a holistic community-based support that is designed for sustainability (Ahmad & Mohamed 2022). In a study conducted among custodial grandparents in Australia, the study revealed the issue of financial burden occasioned by care of the orphans, stating that the grandparents do give up on attending to their personal health need by concentrating on solving the needs of their grandchildren that are orphans in their care. The deduction from the study implied that a holistic view of the support for the grandparent is a panacea to addressing grandparents’ health needs.

This study revealed a need for activation of referrals; some of the respondents stated that grandparents should be referred to the clinics regularly, adolescent (orphans) should be referred to the CYP for counselling and medical support should be provided through complete screening. In a study carried out in China, grandparents with better financial standing are able to cater for their health needs and engage in meaningful social activities (Wang et al. 2020). Grandparents assumed the role of household heads and provide the orphans with basic educational guidance and support, and imbibed the mode of valued custom and practices in the orphans (Lazaro et al. 2023). In a study carried out among the Zimbabwean grandparents, provision of counselling services for the caregiver of the orphans was suggested as the means to enhance the well-being of the grandparents (Mhaka-Mutepfa 2018). The strategy to addressing grandparent’s challenges from the prism of view of the MDT is to put in a place referral to quarters where support could be rendered (Mabena et al. 2024). Among the list stated by the team were constant visit to the clinic for check-ups, referral of the orphans in the care of grandparents for psychoeducational counselling on issues as this could bring succour to the grandparents.

## Conclusion

The study on MDT perception of health needs and support needed for grandparents of children orphaned because of AIDS concluded that the majority of grandparents had no sufficient support structure and most were the heads of household. The study discovered that most of the MDT was aware of the health risks of the grandparents caring for children orphaned because of AIDS. However, the study concluded that there was a lack of income for sustenance, which is contributing to the health problem among grandparents caring for AIDS orphans. It is recommended that the government should see to the plight of grandparents by providing fund to support and to improve their health.
